# Supplementary material for: Integrating single-cell and spatially resolved transcriptomic strategies to survey the astrocyte response to stroke in male mice
Source: Nat Commun. 2024 Feb 21;15:1584. doi: 10.1038/s41467-024-45821-y (PMC10882052; doi:10.1038/s41467-024-45821-y)
Supplement: Supplementary file 1 — Supplementary Information [file 41467_2024_45821_MOESM1_ESM.docx]

**SUPPLEMENTARY INFORMATION**

**Integrating single-cell and spatially resolved transcriptomic strategies to survey astrocytes in response to stroke.**


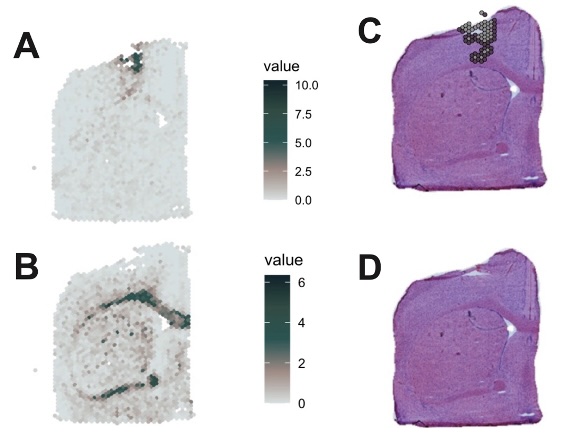


Supplementary Figure 1. **Visium d2 sham section**

(**A & B**) Visium d2 sham sections overlaid with NMF factor 1 (**A**) and factor 2 (**B**) from Figure 2A. The value represents the summed SCT normalized expression values. Individual UMAP clusters associated with the d2 infarct (grey spots, black spots depict border) (**C**) and d10 injury space (**D**) from Figure 2E.

Supplementary Figure 2. **Deconvolution of cell types**.

Visium d2 (top row), d10 (middle row) and d21 (bottom row) sections deconvoluted to show cell types. Visium cortex data from the Allen Brain Institute was used as a reference. The values represent the cell type prediction scores. ENDO=endothelial cells, MACRO=macrophages, ASTRO=astrocytes, OLIGO=oligodendrocytes, VLMC= vascular and leptomeningeal cells, L2/3 IT=Layer 2 projecting to 3 intratelencephalic neurons, L4=Layer 4 neurons, L5 IT= layer 5 intratelencephalic neurons, L6 CT= layer 6 corticothalamic neurons, L6 IT= layer 6 intratelencephalic neurons.


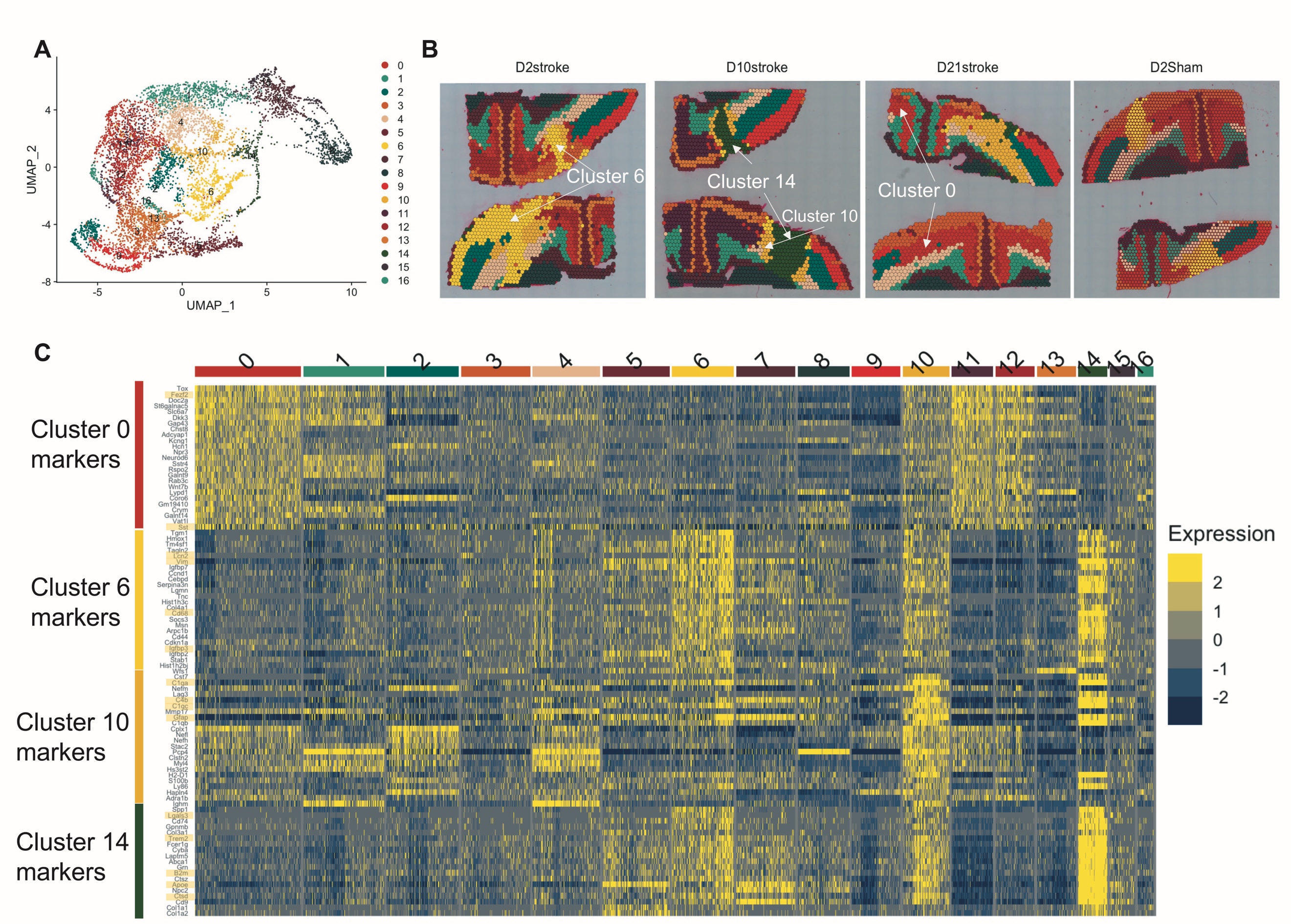


Supplementary Figure 3. **Robust findings across Visium replicates**

(**A**) UMAP plot of 7635 Visium spots from d2 (2163), d10 (1692), d21 (1708) stroke-injured, and d2 sham (2072) samples. n=2 for each time point.

(**B**) Visium d2, d10, d21, and d2 sham sections overlaid with all clusters from (A). At d2 post-stroke, cluster 6 is associated with the infarct. At d10, cluster 14 is associated with the infarct, while cluster 10 is located peripheral to the infarct. At d21 Cluster 0 shows restoration of the cortical layer structure. All indicated by white arrows.

(**C**) A heatmap with the top 25 genes (rows) enriched in clusters 0, 6, 10, and 14, across all spots that are binned into cluster groups (columns). Genes highlighted in yellow appear in Main Figure 2, as well as replicates in (B). The expression represents SCT normalized expression values where high is bright yellow and low is dark purple.

**
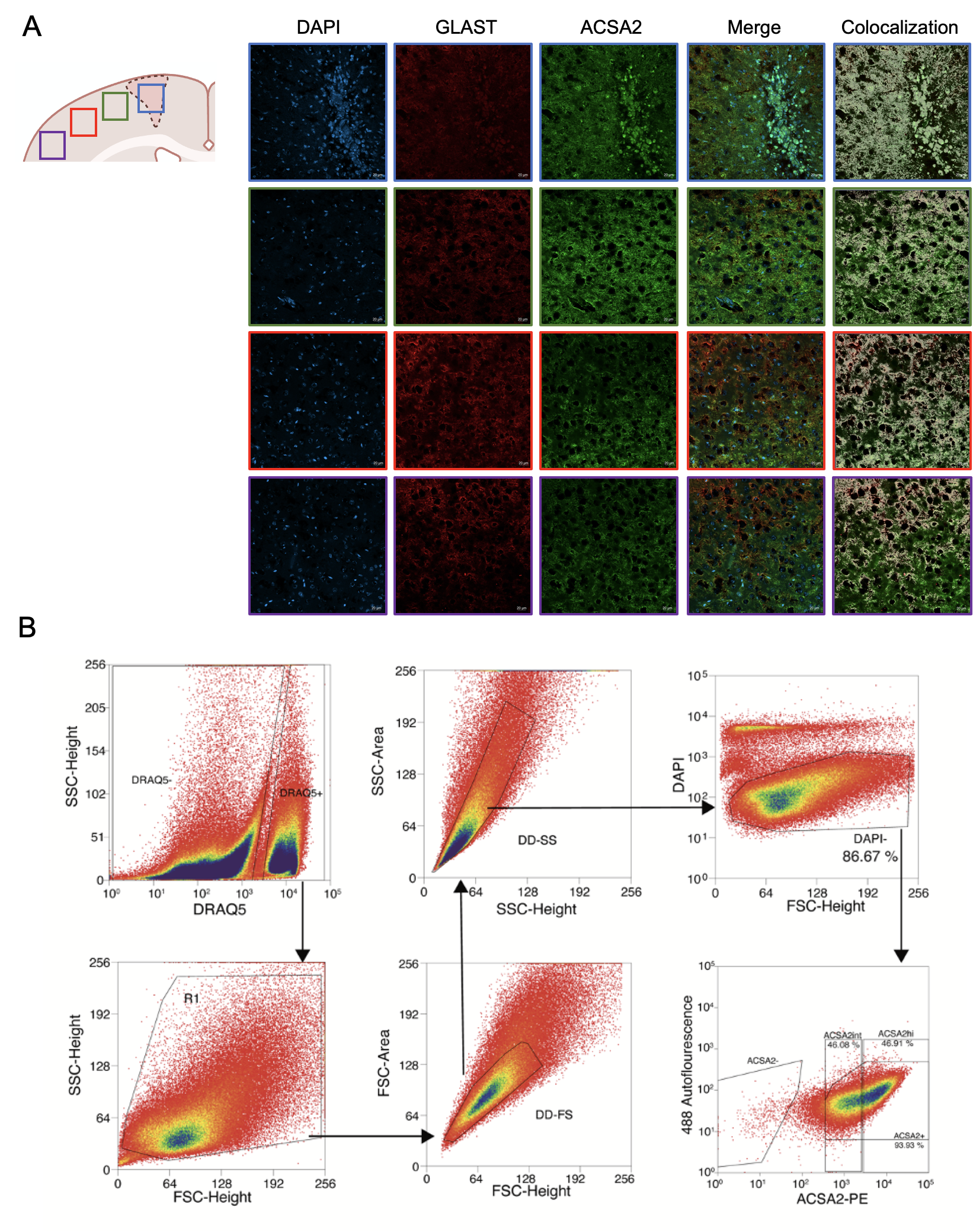
**

Supplementary Figure 4. **FACS isolation of live, nucleated ACSA2+ cells.**

**A**) Astrocytes across the stroke-injured cortex co-express GLAST and ACSA2. Schematic shows location of images within the injured cortex. Colocalization masks are shown in grey. Rows represent increasing distances from the infarct site, and match box colors in schematic. scale bars = 20 µm, n = 3 mice. Biorender was used to create the schematic.

**B**) Flow cytometry gating strategy. DRAQ5+ nucleated cells were identified and gated based on FSC-Height vs. SSC-Height. Doublets were excluded in FSC-Height vs. FSC-Area and SSC-Height vs. SSC-Area gates. Viable DAPI- cells were identified (86.67%) and ACSA2-PE fluorescence intensity was examined. ACSA2+ cells comprised 93.93% of the viable nucleated cells. Within the ACSA2+ fraction, 46.08% of cells exhibited intermediate ACSA2-PE fluorescence intensity (ACSA2int) and 46.91% exhibited high ACSA2-PE (ACSA2hi) fluorescence intensity. This experiment was repeated 3 times with similar results.


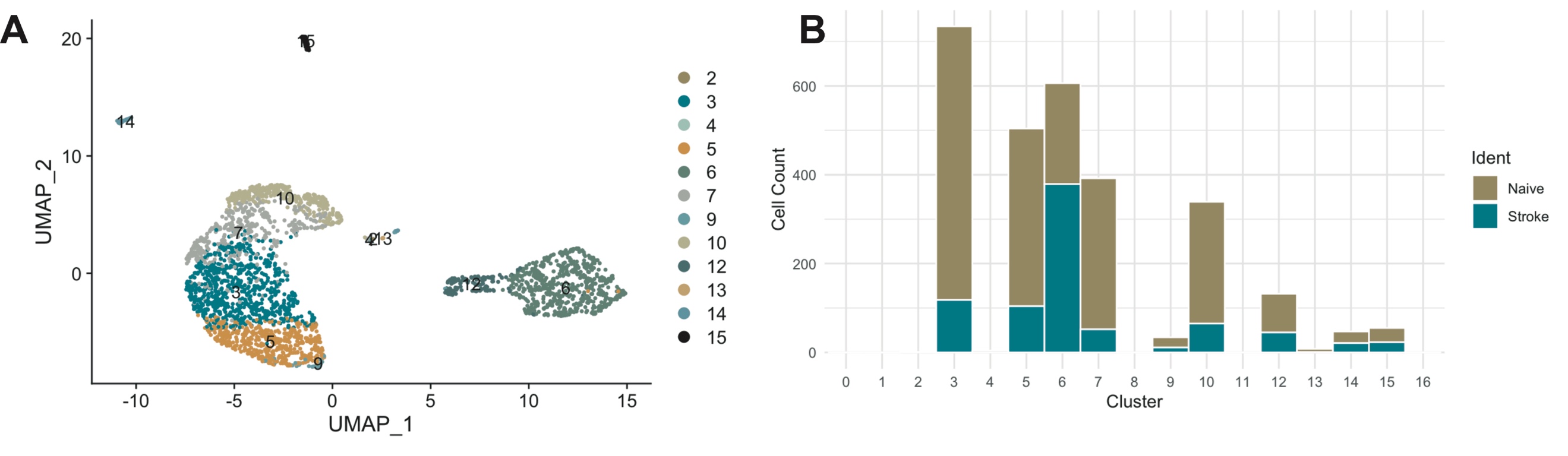


**Supplementary Figure 5.** Subset of astrocyte marker expressing cells**.**

(**A**) UMAP plot of 3521 ACSA-2+ cells from the stroke-injured (1189 cells) and uninjured (2332 cells) cortex. Clusters are numbered according to the clusters in Main Figure 3A.

(**B**) Histogram depicting the number of cells belonging to uninjured (naïve) versus stroke-injured samples across each cluster from (A).

***
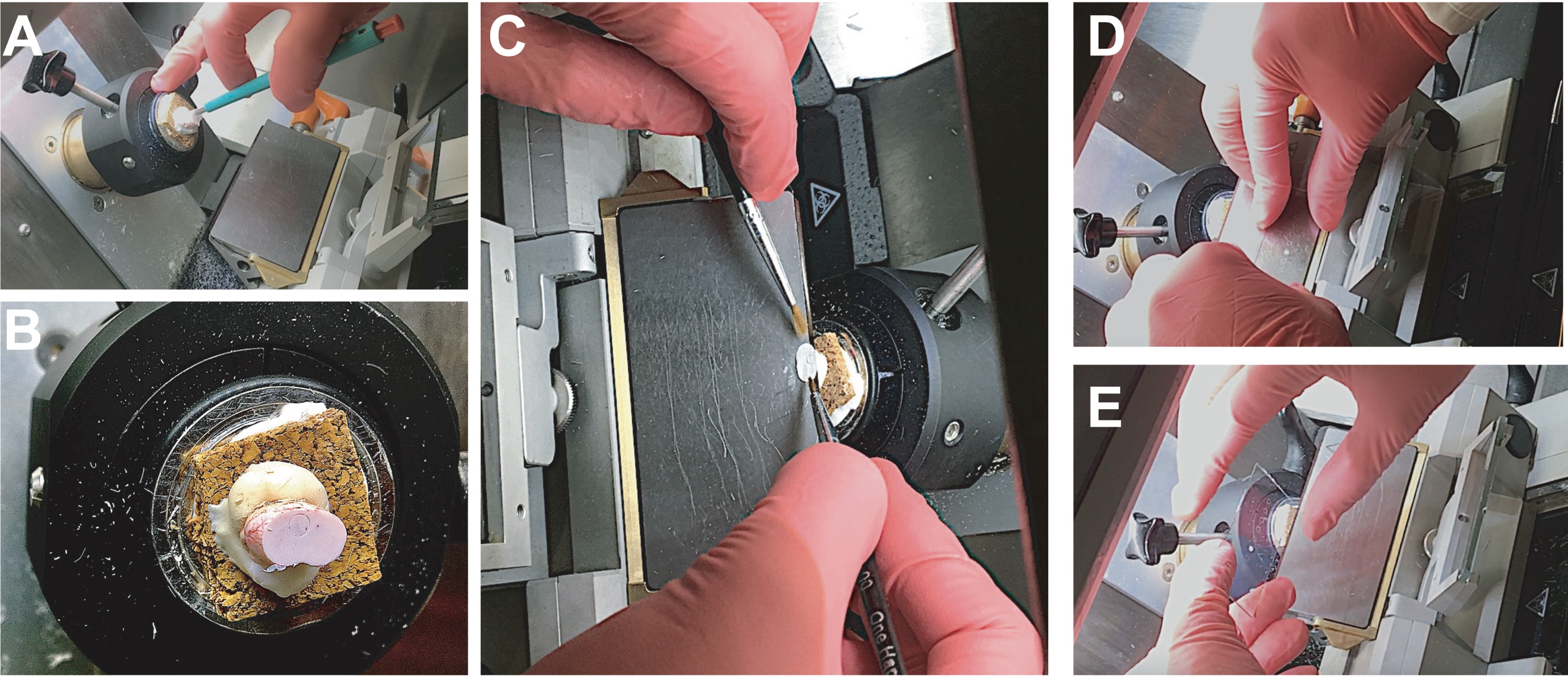
***

Supplementary Figure 6. **Collection of tissue sections for tDISCO.**

Snap frozen tissue is mounted on a cryostat chuck and biopsied with a 2 mm biopsy pen to demarcate a region of interest (**A**, result is **B**). A 10um section (**C**) is collected onto tDISCO top-plate (**D**, result is **E**).


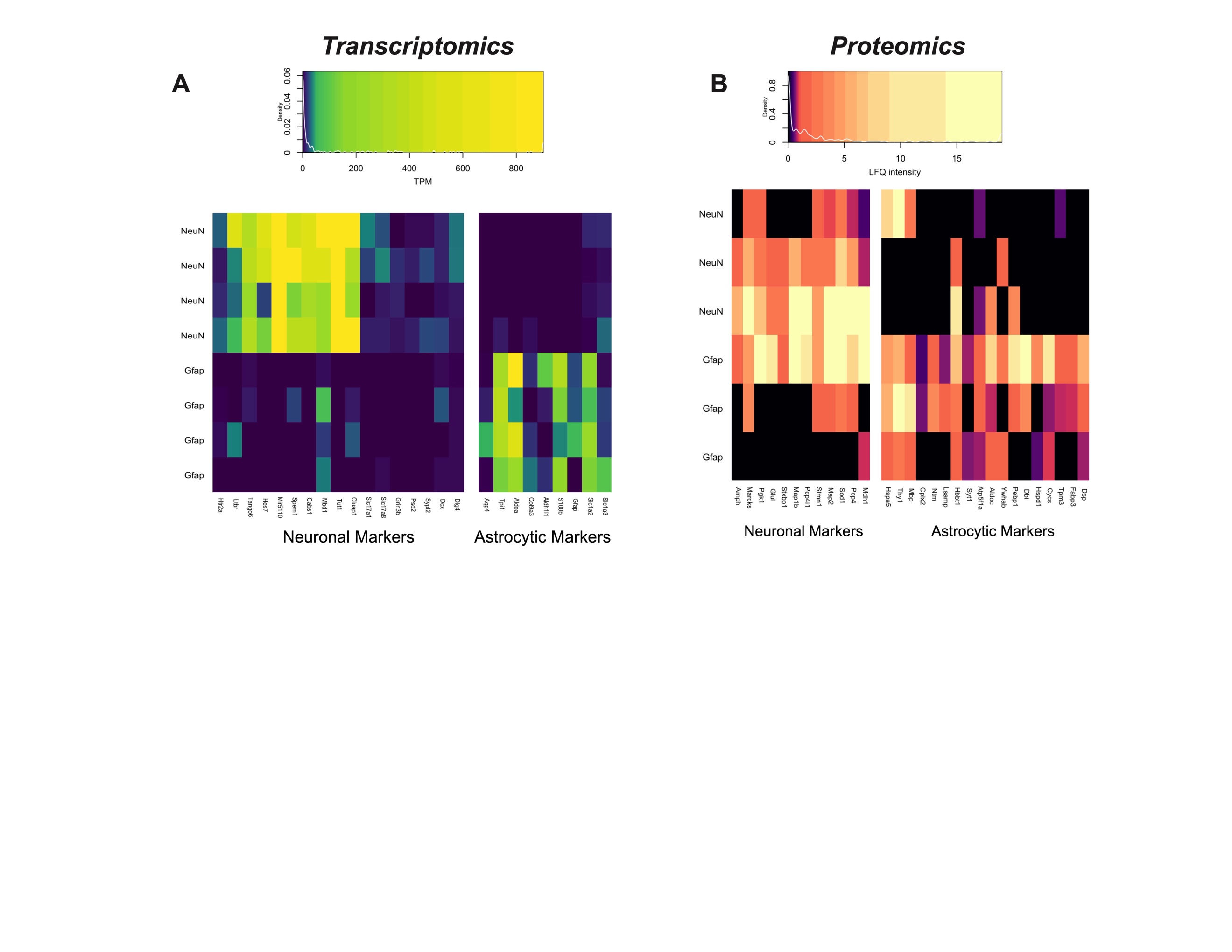


Supplementary Figure 7. **Astrocyte and neuronal gene expression in tDISCO- selected GFAP+ and NEUN+ cells.** Astrocyte and neuronal markers corresponding to Figure 4C-E. (**A**) Transcriptomic data represented in TPM normalized gene expression. n = 4 GFAP+ and 4 NEUN+ cells from across 3 animals (**B**) proteomic data represented as LFQ normalized protein expression n = 3 GFAP+ and 3 NEUN+ cells from across 3 animals.


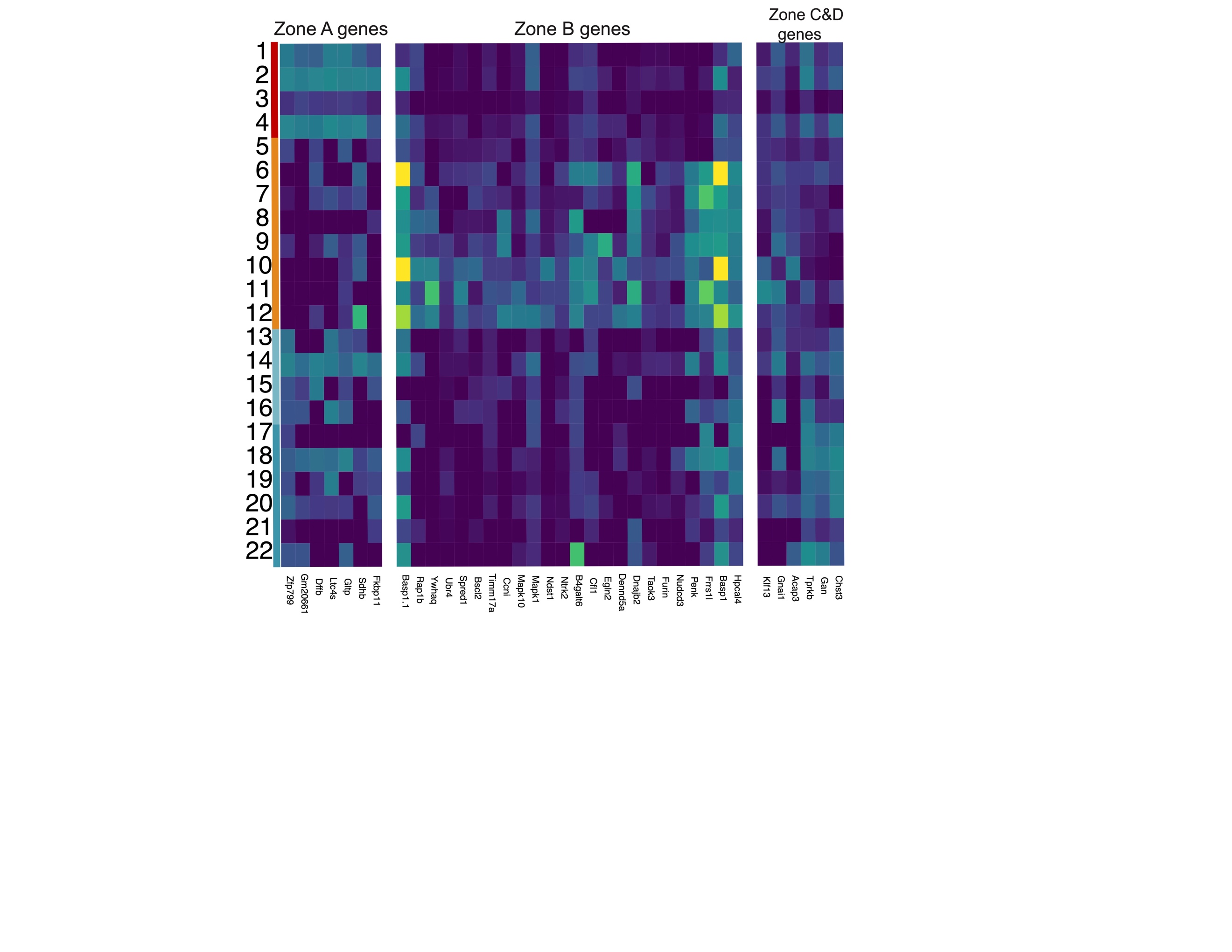


Supplementary Figure 8. **tDISCO detected differential gene expression of zone A proximal astrocytes.** As in Figure 4F, the heatmap depicts numbered GFAP+ cells (1-22, bottom) isolated from zones A, B, and C & D. Gene expression is TPM normalized. n = 22 cells from across 3 animals.


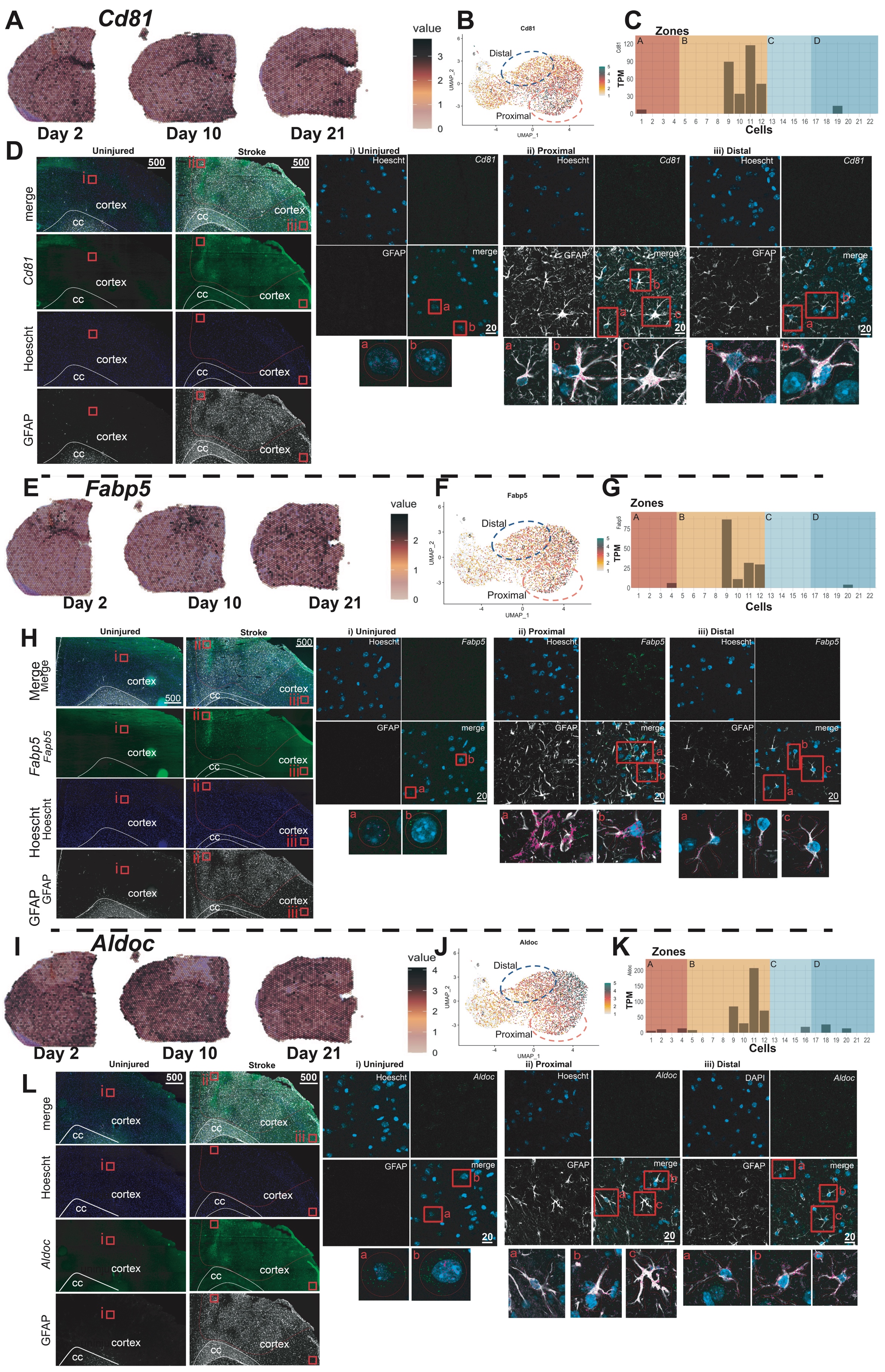


Supplementary Figure 9. **Expression of *Cd81*, *Fabp5*, and *Aldoc* in proximal and distal zones.**

(**A**) Visium d2, d10, and d21 sections show *Cd81* gene expression that is specific to the d10 injury site.
(**B**) UMAP plot of the 10x Chromium scRNAseq data annotated with cluster 6 (peach, distal astrocytes) and cluster 7 (navy blue, proximal astrocytes) shows *Cd81* expression is localized to cluster 7 (proximal) astrocytes.

(**C**) Histogram depicts tDISCO-derived TPM normalized gene expression (y-axis) of *Cd81* in GFAP+ cells (numbered 1-22, x-axis) from zones A (red), B (orange), C (light blue), and D (dark blue).
(**D**) Representative images of *Cd81* and GFAP expression in uninjured (left column) and stroke injured brains (second from left column). The red dotted line in stroke-injured brains denotes the injured area as defined by GFAP staining. Red boxes show the location of the higher magnification images that correspond to uninjured brain (i), and proximal (ii)- and distal (iii)- regions in the stroke-injured brain. Images are at 10x, scale bar = 500 µm. Higher magnification images show *Cd81* and GFAP expression in i) uninjured, (ii) proximal and (iii) distal astrocytes (colocalization is depicted by the pink overlap mask). Images are at 40X, scale bars = 20 µm, n = 3 mice. cc = corpus callosum
(**E-H**) *Fabp5* expression in Visium (H), 10X Chromium (F), and tDISCO (G) datasets. Representative images of *Fabp5* and GFAP expression in uninjured (left column) and stroke-injured brains (second from left column), and in i) uninjured, (ii) proximal and (iii) distal GFAP+ astrocytes (colocalization is depicted by pink overlap mask) (H). Images are at 40X, scale bars = 20 µm, n = 3 mice. cc = corpus callosum.
(**I-L**) *Aldoc* expression in Visium (I), 10X Chromium (J), and tDISCO (K) datasets. Representative images of *Aldoc* and GFAP expression in uninjured (left column) and stroke-injured brains (second from left column), and in i) uninjured, (ii) proximal and (iii) distal GFAP+ astrocytes (colocalization is depicted by pink overlap mask) (L). Images are at 40X, scale bars = 20 µm, n = 3 mice. cc = corpus callosum.

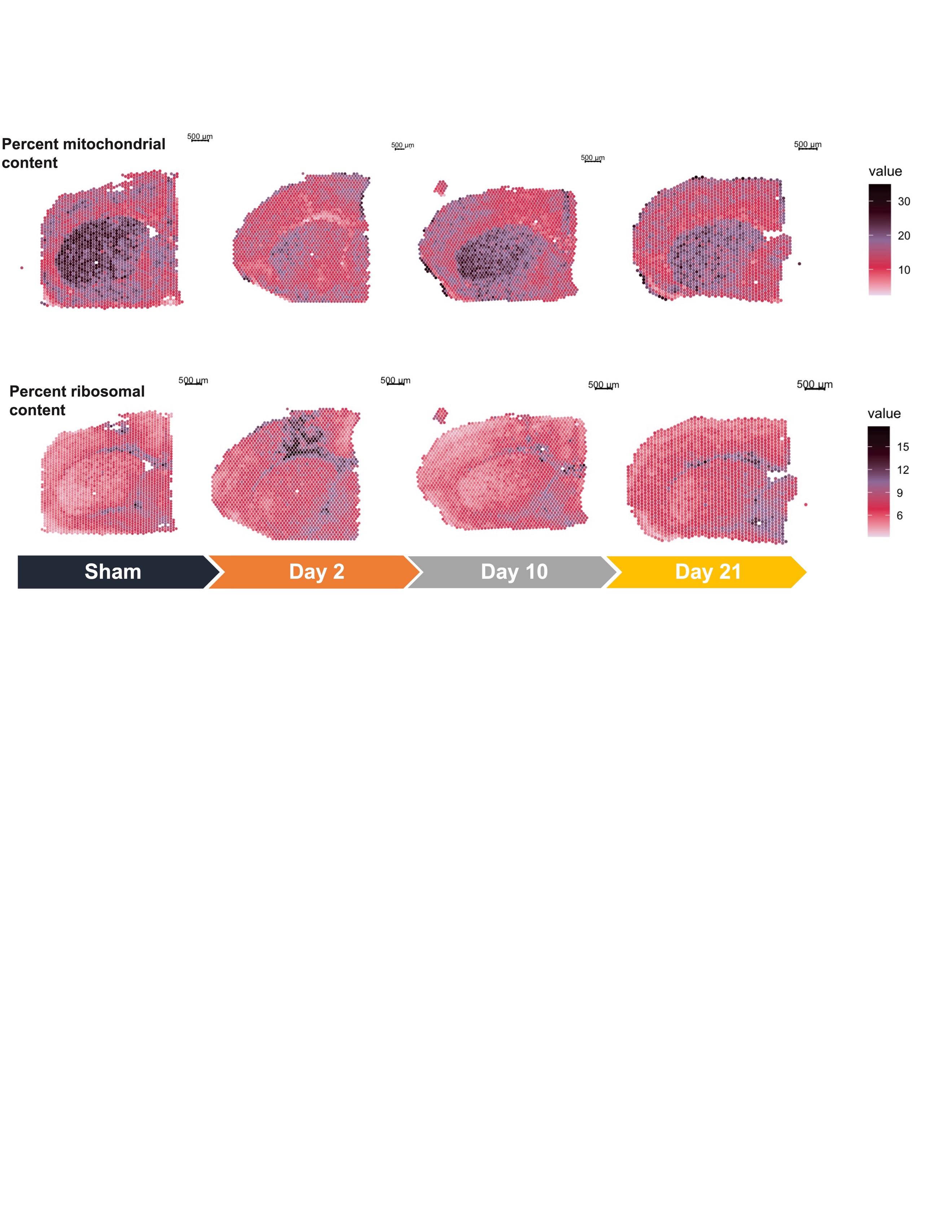


**C**

Supplementary Figure 10**.** **Detection metrics for Visium samples.**

(**A**) the count distribution of the number of genes per spot (top left), the number of counts per spot (top right), the number of UMIs per gene (bottom left), and the number of spots per gene (bottom right)

(**B**) The mean-variance relationship (left) and the mean detection rate relationship (right)
(**C**) The percent mitochondrial content (top) and ribosomal content (bottom) in each section (columns).

Supplementary Figure 11. **Detection metrics for 5695 ACSA-2+ uninjured (UI) and stroke (S) 10X Chromium scRNA-seq samples.**

Stroke (**A**) and uninjured (**B**) datasets. Distribution of the number of genes per cell (left), number of counts per cell (middle), and percent mitochondrial content per cell (right). Each dot represents a cell.

Supplementary Figure 12. **Detection metrics associated with the tDISCO scRNA-seq dataset.** Read (**A**) and gene (**B**) counts across three separate rounds of tDISCO collection, with corresponding gene type percent expression across all samples (**C**) and separated across the three separate rounds (**D**).


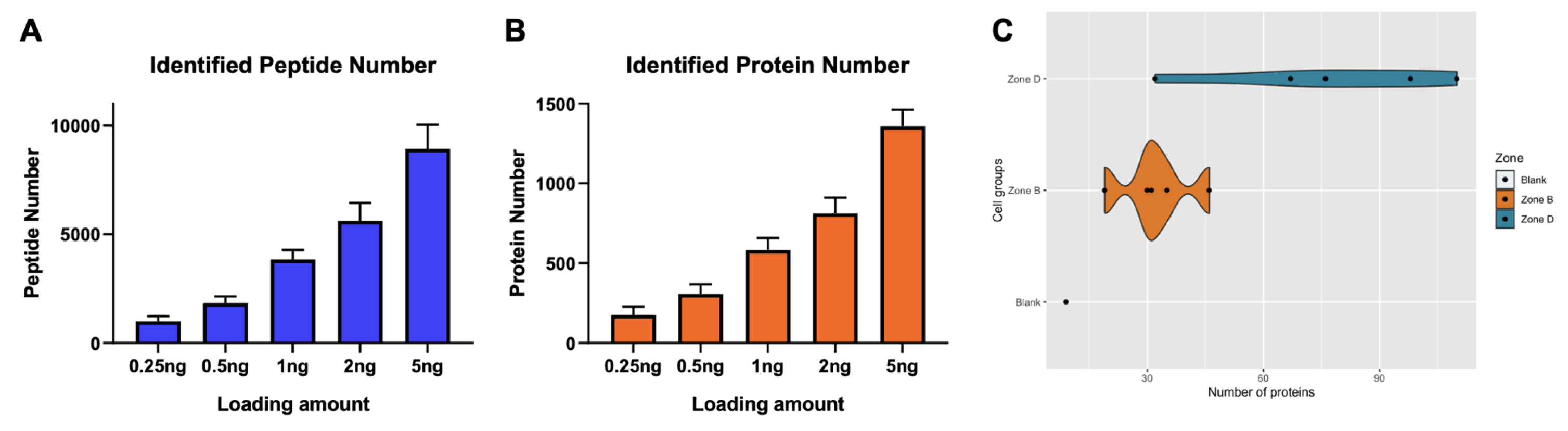


Supplementary Figure 13. **Identification of peptides and proteins from Hela standard solutions as well as from tDISCO derived cell lysates detected by HPLC-MS/MS.** (**A**) Identified peptide numbers from 0.25, 0.5, 1, 2, and 5 ng of Hela digest standard, respectively. (**B**) Identified protein numbers from 0.25, 0.5, 1, 2, and 5 ng of Hela digest standard, respectively. Each experiment was repeated three times. Error bars represent standard deviation. (**C**) The distribution of the number of detected proteins seen across the Zone B cells (5 cells, blue), Zone D cells (5 cells, orange), and the respective blank control for this experiment. The detected proteins on the y-axis had to satisfy presence in 2 or more replicates in each treatment group in order to be considered detected.

**Supplementary Table 1.** Sequences of primers used for tDISCO RT and sequencing

| Name | Sequence |
| --- | --- |
| Cell Barcode 1 | /5Biosg/TTTTTAAGC+AGTGGT+ATCAAC+GCAGAGTA+CNNNNN+TAGCTCGTGCCT +GNNNNNNNNTTTTTTTTTTTTTTTTTTTTTTTTTTTTTTV |
| Cell Barcode 2 | /5Biosg/TTTTTAAGC+AGTGGT+ATCAAC+GCAGAGTA+CNNNNNNN+CTATGTGTCAC+ANNNNNNNNTTTTTTTTTTTTTTTTTTTTTTTTTTTTTTV |
| Cell Barcode 3 | /5Biosg/TTTTTAAGC+AGTGGT+ATCAAC+GCAGAGTA+CNNNNNNNN+GCGCGTAG+CTTGNNNNNNNNTTTTTTTTTTTTTTTTTTTTTTTTTTTTTTV |
| Cell Barcode 4 | /5Biosg/TTTTTAAGC+AGTGGT+ATCAAC+GCAGAGTA+CNNNNNNNNN+GCACACCTGC+TCNNNNNNNNTTTTTTTTTTTTTTTTTTTTTTTTTTTTTTV |
| Cell Barcode 5 | /5Biosg/TTTTTAAGC+AGTGGT+ATCAAC+GCAGAGTA+CNNNNNNNNN+AGACATGAGA+GTNNNNNNNNTTTTTTTTTTTTTTTTTTTTTTTTTTTTTTV |
| Cell Barcode 6 | /5Biosg/TTTTTAAGC+AGTGGT+ATCAAC+GCAGAGTA+CNNNNN TA+GCTCGTGCC+TNNNNNNNNTTTTTTTTTTTTTTTTTTTTTTTTTTTTTTV |
| Cell Barcode 7 | /5Biosg/TTTTTAAGC+AGTGGT+ATCAAC+GCAGAGTA+CNNNNNNN+TATGAGACTCT+ANNNNNNNNTTTTTTTTTTTTTTTTTTTTTTTTTTTTTTV |
| Cell Barcode 8 | /5Biosg/TTTTTAAGC+AGTGGT+ATCAAC+GCAGAGTA+CNNNNNNNN+GATATAGCT+CTANNNNNNNNTTTTTTTTTTTTTTTTTTTTTTTTTTTTTTV |
| Cell Barcode 9 | /5Biosg/TTTTTAAGC+AGTGGT+ATCAAC+GCAGAGTA+CNNNNNNNNN+CGATGATC+GTATNNNNNNNNTTTTTTTTTTTTTTTTTTTTTTTTTTTTTTV |
| Cell Barcode 10 | /5Biosg/TTTTTAAGC+AGTGGT+ATCAAC+GCAGAGTA+CNNNNNNNNN+CCAGACTCACT+CNNNNNNNNTTTTTTTTTTTTTTTTTTTTTTTTTTTTTTV |
| TSO | AAGCAGTGGTATCAACGCAGAGTACrGrGrG |
| Index Primer 1 | /5Biosg/GGCATGAGTGGCTGCTAGATCGCGCGGAAGCAGTGGTATCAACGCAGAGT |
| Index Primer 2 | /5Biosg/GGCATGAGTGGCTGCCTCTCTATGCGGAAGCAGTGGTATCAACGCAGAGT |
| Index Primer 3 | /5Biosg/GGCATGAGTGGCTGCTATCCTCTGCGGAAGCAGTGGTATCAACGCAGAGT |
| Index Primer 4 | /5Biosg/GGCATGAGTGGCTGCAGAGTAGAGCGGAAGCAGTGGTATCAACGCAGAGT |
| Index Primer 5 | /5Biosg/GGCATGAGTGGCTGCGTAAGGAGGCGGAAGCAGTGGTATCAACGCAGAGT |
| Custom P5 oligo | AATGATACGGCGACCACCGAGATCTACACGACTCTAGGGCATGAGTGGCT*G*C |
| Custom Read1 primer | GCGGAAGCAGTGGTATCAACGCAGAGTAC |
| Custom Index 2 primer | GACTCTAGGGCATGAGTGGCTGC |
