# Supplementary material for: Integrating single-cell and spatially resolved transcriptomic strategies to survey the astrocyte response to stroke in male mice
Source: Nat Commun. 2024 Feb 21;15:1584. doi: 10.1038/s41467-024-45821-y (PMC10882052; doi:10.1038/s41467-024-45821-y)
Supplement: Supplementary file 3 — Description of Additional Supplementary Files [file 41467_2024_45821_MOESM3_ESM.docx]

**Description of Additional Supplementary Files**

**Supplementary Data 1:**

Genes enriched in the astrocytes from the putatively proximal [to the stroke injury site] cluster 6 from Figure 3C (main text). These genes were obtained using FindMarkers function from Seurat.
