# Supplementary material for: Integrating single-cell and spatially resolved transcriptomic strategies to survey the astrocyte response to stroke in male mice
Source: Nat Commun. 2024 Feb 21;15:1584. doi: 10.1038/s41467-024-45821-y (PMC10882052; doi:10.1038/s41467-024-45821-y)
Supplement: Supplementary file 5 — Reporting Summary [file 41467_2024_45821_MOESM5_ESM.pdf]

nature portfolio

Corresponding author(s): Maryam Faiz
Last updated by author(s): Jan 15, 2024

Reporting Summary

Nature Portfolio wishes to improve the reproducibility of the work that we publish. This form provides structure for consistency and transparency in reporting. For further information on Nature Portfolio policies, see our Editorial Policies and the Editorial Policy Checklist.

Statistics

For all statistical analyses, confirm that the following items are present in the figure legend, table legend, main text, or Methods section.

n/a	Confirmed	
☐	☒	The exact sample size (*n*) for each experimental group/condition, given as a discrete number and unit of measurement
☐	☒	A statement on whether measurements were taken from distinct samples or whether the same sample was measured repeatedly
☒	☐	The statistical test(s) used AND whether they are one- or two-sided<br>*Only common tests should be described solely by name; describe more complex techniques in the Methods section.*
☒	☐	A description of all covariates tested
☐	☒	A description of any assumptions or corrections, such as tests of normality and adjustment for multiple comparisons
☐	☒	A full description of the statistical parameters including central tendency (e.g. means) or other basic estimates (e.g. regression coefficient) AND variation (e.g. standard deviation) or associated estimates of uncertainty (e.g. confidence intervals)
☐	☒	For null hypothesis testing, the test statistic (e.g. *F*, *t*, *r*) with confidence intervals, effect sizes, degrees of freedom and *P* value noted<br>*Give P values as exact values whenever suitable.*
☒	☐	For Bayesian analysis, information on the choice of priors and Markov chain Monte Carlo settings
☐	☒	For hierarchical and complex designs, identification of the appropriate level for tests and full reporting of outcomes
☒	☐	Estimates of effect sizes (e.g. Cohen's *d*, Pearson's *r*), indicating how they were calculated

Our web collection on statistics for biologists contains articles on many of the points above.

Software and code

Policy information about availability of computer code

Data collection	All custom codes used to collect data are available at http://microfluidics.utoronto.ca/gitlab/DISCO.
Data analysis	The codes for analysis are available at https://github.com/eyscott/Visium_10XChromium_tDISCO.

For manuscripts utilizing custom algorithms or software that are central to the research but not yet described in published literature, software must be made available to editors and reviewers. We strongly encourage code deposition in a community repository (e.g. GitHub). See the Nature Portfolio guidelines for submitting code & software for further information.

Data

Policy information about availability of data

All manuscripts must include a data availability statement. This statement should provide the following information, where applicable:

- Accession codes, unique identifiers, or web links for publicly available datasets
- A description of any restrictions on data availability
- For clinical datasets or third party data, please ensure that the statement adheres to our policy

The sequencing and proteomic data generated in this study have been deposited in the NCBI SRA database under Bioproject number PRJNA952594 (https://dataview.ncbi.nlm.nih.gov/object/PRJNA952594?reviewer=k4bk4puu65vkcq0qn0bv9g3ni6) and in ProteomeXchange under accession code PXD041388. The processed sequencing and proteomics data, as well as the code used to process them are available at https://github.com/eyscott/Visium_10XChromium_tDISCO (DOI: 10.5072/zenodo.20771). The source data generated in this study are provided in the Supplementary Information/Source Data file.

Human research participants

Policy information about studies involving human research participants and Sex and Gender in Research.

Reporting on sex and gender	N/A
Population characteristics	N/A
Recruitment	N/A
Ethics oversight	N/A

Note that full information on the approval of the study protocol must also be provided in the manuscript.

Field-specific reporting

Please select the one below that is the best fit for your research. If you are not sure, read the appropriate sections before making your selection.

[x] Life sciences [ ] Behavioural & social sciences [ ] Ecological, evolutionary & environmental sciences

For a reference copy of the document with all sections, see nature.com/documents/nr-reporting-summary-flat.pdf

Life sciences study design

All studies must disclose on these points even when the disclosure is negative.

Sample size	n= 3 animals per condition for 10X Visium. An n=3 sample size was based on current recommendations by 10X Visium (2 replicates; https://kb.10xgenomics.com/hc/en-us/articles/360036298191-Do-we-recommend-running-replicate-tissue-sections-with-Visium-for-fresh-frozen-)<br><br>n = 19,212 cells across two independent 10X Chromium experiments. First experiment = Total of 13,517 cells; 6243 cells from a pool of 12 stroke-injured animals and 7274 cells from a pool of 12 uninjured animals. Second experiment =Total of 5695 cells; 2603 cells from a a pool of 8 stroke-injured animals and 3092 cells from a pool of 8 uninjured animals). Biological pools of 10+ animals per experiment were chosen as there is an upper technical limit on the number of astrocytes that can be sorted from a single stroke-injured cortex. To address concerns related to randomness 2 pools were run.<br><br>n = 22 cells across 3 animals for sc-transcriptomics; 10 cells across 3 animals for sc- proteomics Rationale: technical limits of the evDISCO system.
Data exclusions	Individual cells were excluded that showed either low gene count or high mitochondrial gene content , according to standard single cell -omics workflows.
Replication	Results were replicated across three different platforms. Validation experiments performed with RNAScope were used to supplement the initial findings.
Randomization	Males were randomly allocation to stroke and uninjured groups
Blinding	Blinding is n/a for these experiments.

Reporting for specific materials, systems and methods

We require information from authors about some types of materials, experimental systems and methods used in many studies. Here, indicate whether each material, system or method listed is relevant to your study. If you are not sure if a list item applies to your research, read the appropriate section before selecting a response.

Materials & experimental systems

n/a	Involved in the study	
[ ]	[x]	Antibodies
[x]	[ ]	Eukaryotic cell lines
[ ]	[ ]	Palaeontology and archaeology
[ ]	[x]	Animals and other organisms
[x]	[ ]	Clinical data
[x]	[ ]	Dual use research of concern

Methods

n/a	Involved in the study	
[x]	[ ]	ChIP-seq
[x]	[ ]	Flow cytometry
[x]	[ ]	MRI-based neuroimaging

Antibodies

Antibodies used	Primary antibodies:<br>ACSA2-microbeads (Miltenyi Biotec, 130-097-679, 1:10); ACSA2-PE (Miltenyi Biotec, 130-116-244, 1:100, clone: IH3-18A3); GFAP (AvesLabs, AB_2313547, 1:100, polyclonal); NeuN (EMD Millipore, ABN78, 1:100, polyclonal); Rabbit anti-GFAP (Dako; Z0334, 1:5000, polyclonal)<br><br>Secondary antibodies:<br>Goat anti-chicken IgY, Alexafluor 488 (Invitrogen, A11039, polyclonal, 1:100); Goat anti Rabbit IgG Alexafluor 647 (Invitrogen, A32733, polyclonal, 1:100): Donkey anti-rabbit Alexa Fluor 647 (Jackson Immunoresearch, AB_2492288, polyclonal, 1:500)
Validation	Primary antibodies were validated in negative (1a absent) and positive control tissue and optimized using serial dilution. These antibodies have also been extensively validated by manufacturers showing specificity towards these proteins in the brain for our application of choice (immunohistochemistry or MACS). A list of references citing the usage of this antibody are found on the manufacturer's website.

Palaeontology and Archaeology

Specimen provenance	*Provide provenance information for specimens and describe permits that were obtained for the work (including the name of the issuing authority, the date of issue, and any identifying information). Permits should encompass collection and, where applicable, export.*
Specimen deposition	*Indicate where the specimens have been deposited to permit free access by other researchers.*
Dating methods	*If new dates are provided, describe how they were obtained (e.g. collection, storage, sample pretreatment and measurement), where they were obtained (i.e. lab name), the calibration program and the protocol for quality assurance OR state that no new dates are provided.*

☐ Tick this box to confirm that the raw and calibrated dates are available in the paper or in Supplementary Information.

Ethics oversight	*Identify the organization(s) that approved or provided guidance on the study protocol, OR state that no ethical approval or guidance was required and explain why not.*

Note that full information on the approval of the study protocol must also be provided in the manuscript.

Animals and other research organisms

Policy information about studies involving animals; ARRIVE guidelines recommended for reporting animal research, and Sex and Gender in Research

Laboratory animals	Male C57Bl/6J, 8-10 weeks old
Wild animals	*Provide details on animals observed in or captured in the field; report species and age where possible. Describe how animals were caught and transported and what happened to captive animals after the study (if killed, explain why and describe method; if released, say where and when) OR state that the study did not involve wild animals.*
Reporting on sex	Findings only apply to males. No analysis of sex was carried out in this first study.
Field-collected samples	*For laboratory work with field-collected samples, describe all relevant parameters such as housing, maintenance, temperature, photoperiod and end-of-experiment protocol OR state that the study did not involve samples collected from the field.*
Ethics oversight	All animal protocols were approved by the Animal Care Committees at the Toronto Centre for Phenogenomics (26-0392H) and the Division of Comparative Medicine of the University of Toronto (01155335).

Note that full information on the approval of the study protocol must also be provided in the manuscript.
